# The Impact of Cognitive Behavioral Therapy for Insomnia on Neurofilament Light and Phosphorylated Tau in Individuals with a Concussion

**DOI:** 10.1093/arclin/acae096

**Published:** 2024-11-06

**Authors:** Rebecca Ludwig, Michael Rippee, Linda D’Silva, Jeff Radel, Aaron M Eakman, Jill Morris, Alvin Beltramo, Michelle Drerup, Catherine Siengsukon

**Affiliations:** Department of Physical Therapy, Rehabilitation Science, and Athletic Training, University of Kansas Medical Center, 3901 Rainbow Blvd. Mail Stop 2002, Kansas City, KS 66160, USA; Department of Neurology, University of Kansas Medical Center, 3901 Rainbow Blvd, Mailstop 2012, Kansas City, KS 66160, USA; Department of Physical Therapy, Rehabilitation Science, and Athletic Training, University of Kansas Medical Center, 3901 Rainbow Blvd. Mail Stop 2002, Kansas City, KS 66160, USA; Department of Occupational Therapy and Therapeutic Science, University of Kansas Medical Center, 3901 Rainbow Blvd Mail Stop 2003 Kansas City, KS 66160, USA; Department of Occupational Therapy, Colorado State University, 850 Oval Drive Mail Stop 1501, Fort Collins, CO 80523, USA; Department of Neurology, University of Kansas Medical Center, 3901 Rainbow Blvd, Mailstop 2012, Kansas City, KS 66160, USA; Department of Biostatistics and Data Science, University of Kansas Medical Center, 3901 Rainbow Blvd, Mailstop 1026, Kansas City, KS 66160, USA; Sleep Disorders Center, Cleveland Clinic, Neurological Institute, 9500 Euclid Ave Cleveland, OH 44195, USA; Department of Physical Therapy, Rehabilitation Science, and Athletic Training, University of Kansas Medical Center, 3901 Rainbow Blvd. Mail Stop 2002, Kansas City, KS 66160, USA

**Keywords:** Concussion, NfL, pTau181, Cognitive Behavioral therapy for insomnia, Insomnia

## Abstract

**Background:**

Concussions damage neurologic tissue, increasing release of intercellular proteins including phosphorylated Tau (pTau) and neurofilament light (NfL). Disrupted sleep from a concussion negatively impacts the ability of the glymphatic system to remove cellular waste from the brain.

**Objective:**

The purpose of this study was to determine if enhancing sleep using Cognitive Behavioral Therapy for Insomnia (CBT-I) impacts pTau and NFL levels following a concussion.

**Methods:**

This is pre/post intervention analysis of a larger wait-list control study. Participants had their blood sampled pre/post the CBT-I intervention which was analyzed using SIMOA analytics. Paired sampling statistics and linear regression models were used to examine how insomnia severity impacts pTau181 and NfL.

**Results:**

Twenty-eight participants were enrolled in this study. Age and baseline protein level were significantly associated with post-intervention protein levels, but post-intervention insomnia severity was not associated with post-intervention protein levels. About 50% of participants that had clinically meaningful change in insomnia and had a reduction in their NfL and pTau181 values.

**Conclusions:**

Post-intervention insomnia was not associated with post-intervention NfL or pTau. Yet, on an individual level, ~50% of participants had a clinically meaningful change in insomnia and reduced level of NfL and pTau 18.1.

**Clinical Trial Registration:**

NCT04885205 https://clinicaltrials.gov

## INTRODUCTION

A concussion is caused by biomechanical forces placed on the head and body that induces a myriad of symptoms such as a headache, loss of balance, or sleep disturbance; costing upwards of $40.6 billion per year between direct and indirect healthcare costs (Chancellor et al., 2019; Miller et al., 2021). The forces twist and shear neurologic tissue, resulting in diffuse axonal injury, and release of intercellular cytoskeleton proteins including Tau and neurofilament light (NfL) (Frati et al., 2017; Ma et al., 2016; Senaratne et al., 2022; Smith et al., 2003). The role of NfL and Tau is to support and maintain the structural integrity of the myelinated and non-myelinated axons, respectively (Zetterberg et al., 2013). Higher levels of serum NfL and Tau indicate neuronal damage remains months to even years following the concussion injury, possibly contributing to prolonged recovery (McDonald et al., 2021; Rubin et al., 2019; Shahim et al., 2016; Shahim et al., 2018; Shahim et al., 2020b).

Poor sleep, defined as decreased quality or quantity of sleep, is a common symptom following a concussion and has also been associated with prolonged recovery (Ludwig et al., 2020; Ludwig et al., 2021; Wiseman-Hakes et al., 2022). In individuals with a concussion, insomnia is the most reported sleep disturbance, occurring in ~50% of individuals (Ouellet et al., 2015). The factors leading to insomnia are generally multifactorial in individuals post-concussion (Morin et al., 2006; Perlis et al., 1997; Sateia, 2014). The axonal damage from the concussion can cause dysregulation of the sleep and arousal centers contributing to insomnia (Ludwig et al., 2020; Mollayeva et al., 2016; Parcell et al., 2008; Singh et al., 2016; Wickwire et al., 2016). Insomnia can also result from secondary factors such as medications, anxiety, depression, change in routine and sleep schedule, as well as the added stress of post-concussion symptom recovery (Ayalon et al., 2007; Ludwig et al., 2020; Zumstein et al., 2011).

Poor sleep, particularly the loss of slow wave sleep, can impair the glymphatic system which is crucial for clearance of metabolic waste including NfL and Tau (Jessen et al., 2015; Jin et al., 2024; Reddy & van der Werf, 2020). Individuals with chronic insomnia are at an increased risk for decreased glymphatic system function (Jin et al., 2024). In one retrospective chart review, individuals who had a concussion and poor sleep quality had elevated levels of plasma NfL and total Tau compared to individuals who experienced good sleep quality and did not have a concussion (Werner et al., 2020). The glymphatic system functions optimally during sleep, cleansing the brain of neurotoxins and metabolites that accumulate from cellular processes (Jessen et al., 2015; Mendelsohn & Larrick, 2013; Reddy & van der Werf, 2020; Xie et al., 2013). However, when slow-wave sleep is limited, as often occurs in individuals with insomnia, the glymphatic system remains suppressed, leading to the accumulation of neurotoxins in the brain (Reddy & van der Werf, 2020). This suppression, when combined with a concussion injury, may significantly disrupt glymphatic function, leading to the build-up of these toxins, potentially resulting in prolonged recovery and increasing the risk of developing neurodegenerative diseases (Jessen et al., 2015; Jin et al., 2024; Reddy & van der Werf, 2020).

Cognitive Behavioral Therapy for Insomnia (CBT-I) is the first line treatment for insomnia and is superior to pharmaceutical interventions (Edinger & Means, 2005). CBT-I is a multicomponent program that addresses the perpetuating factors of insomnia through cognitive restructuring, relaxation training, developing appropriate coping strategies, and utilizing behavioral techniques to combat insomnia and is superior to stand alone sleep hygiene techniques (Edinger et al., 2009; Edinger & Means, 2005). CBT-I has been effectively used to improve sleep outcomes in the general population and in people with neurological injury or disease (Ludwig et al., 2020; Mitchell et al., 2012; Patel et al., 2017; Siengsukon et al., 2020; Trauer et al., 2015), but there is limited evidence about the efficacy of CBT-I in individuals with a concussion. There is only one completed study utilizing CBT-I in individuals with a concussion (Tomfohr-Madsen et al., 2019). The results of this study indicated that the CBT-I intervention improved sleep outcomes but did not address if improved sleep impacted post-concussion symptoms or cytoskeleton proteins.

If an individual with a concussion is not sleeping well, both cytoskeleton proteins and normal metabolic waste can accumulate because the glymphatic system is not functioning optimally (Jessen et al., 2015; Reddy & van der Werf, 2020). Enhancing sleep may improve clearance of metabolites and cytoskeleton proteins. Therefore, the purpose of this study is to evaluate the effect of CBT-I on levels of NfL and phosphorylated tau 181 (pTau181) biomarkers in individuals with a subacute concussion and symptoms of insomnia. It is hypothesized there will be a significant reduction in plasma NfL and pTau181 from baseline to post-CBT-I. Additionally, this study explored how change in insomnia predicts change in NfL and pTau181. Lastly, this study explored how participant improvements in insomnia scores and reduction of protein level could be dichotomized.

## METHODS

This is the exploratory aim of a larger randomized clinical trial and is a pilot pre/post analysis evaluating the before and after results of NfL and pTau181 proteins following the CBT-I intervention (Ludwig et al., 2024). In-depth methods can be found in the protocol manuscript and full details regarding improvement in sleep scores, symptom burden and severity, and co-morbid anxiety and depression can be found in the main study paper (Ludwig et al., 2022; Ludwig et al., 2024). Pertinent methods for this study are described below. The Institutional Review Board of the University of Kansas Medical Center approved the study in accordance with the Declaration of Helsinki. All participants completed an informed consent process.

### Research design

**Fig. 1:**
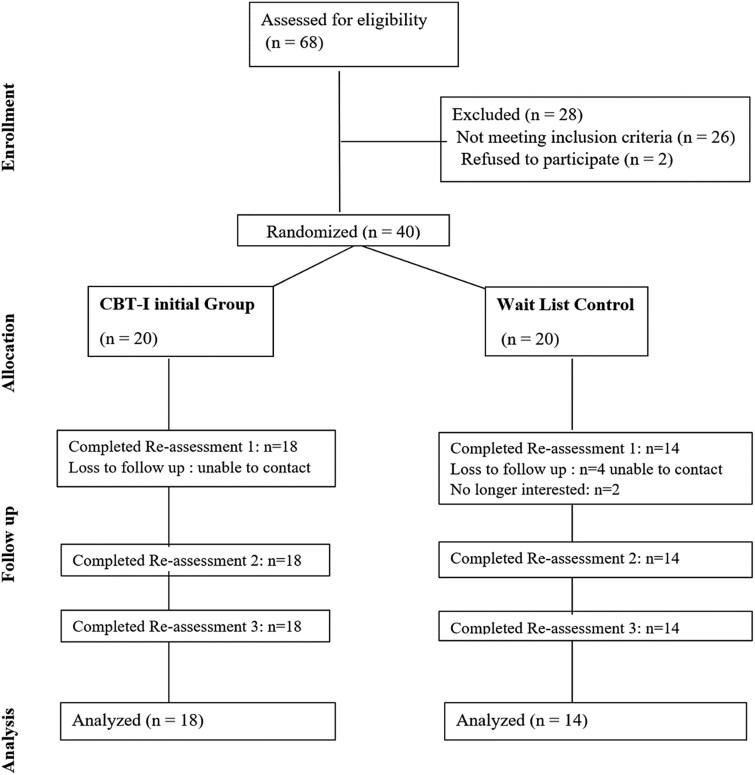
CONSOT diagram.

Individuals with a concussion were enrolled in the parent randomized control waitlist design study and randomized into either a group that started CBT-I initially (n = 20) after baseline or a group that waited 6 weeks before starting the CBT- I (n = 20) (Fig. 1). The CBT-I sessions were done once a week for 6 weeks. During the 6 weeks of waiting for the CBT-I intervention, individuals were instructed to maintain their typical daily activities and medical/therapy appointments. For this study, participants are in one group for the pre/post analyses.

### Inclusion/exclusion criteria

Individuals met inclusion criteria if they were (i) between the ages of 18 and 64 years old; (ii) were at least 4 weeks post their physician-diagnosed concussion injury; (iii) scored greater than 10 on the Insomnia Severity Index, (Bastien et al., 2001) and; (iv) did not have a history of a sleep disorder other than insomnia. Individuals were excluded if they (i) had a history of a another neurologic disorder; (ii) severe depression (greater than 30 on the Beck Depression Inventory [BDI] [Beck et al., 1961]); (iii) were at risk for suicide as indicated by the BDI or; (iv) worked the night shift.

### Blood draw procedures

Individuals who met eligibility requirements had their blood drawn as part of baseline assessments, 1 week or 6 weeks before the CBT-I intervention depending on group allocation. Participants then had their follow-up blood draw within 1 week of completing the CBT-I intervention.

The blood was drawn (~10 ml) by a trained phlebotomist into a vacutainer tube containing ethylenediaminetetraacetic acid (EDTA) as an anti-coagulant. The EDTA tube was then inverted several times to mix the anti-coagulant and placed in a centrifuge at 1500 g for 10 min at 4 degrees Celsius. The plasma was then transferred into aliquots and stored in a − 80 degree Celsius freezer until analysis. NfL and pTau181 analyses were performed on a Single Molecule Array (SIMOA) HD-X analyzer (Quanterix, Billerica, MS, USA) per manufacturer instructions. Analyses were performed on the first freeze–thaw of the samples.

### Statistics

Descriptive statistics (means, standard deviation, and percentages) were used to describe the demographics of the sample. Wilcoxon Signed Rank tests were used to evaluate the change in biomarker level from before to after CBT-I intervention. Within-group effect sizes for non-parametric were calculated to assess magnitude of change and were interpreted as small <0.10–0.29; medium <0.30–0.49; and large ≥0.50. (Kraemer & Andrews, 1982; Lötsch & Ultsch, 2020; Tomczak & Tomczak, 2014) Linear regression analyses were performed with post intervention NfL (Model 1) and pTau181 (Model 3) values as the dependent variable and post intervention ISI values as the independent variable. Next, in Model 2 and 4, post intervention NfL (Model 2) and pTau181 (Model 4) values were the dependent variable and post intervention ISI scores was the independent variables with moderators of age, sex, and baseline protein levels included. It is well known that increase in age is associated with higher levels of both NfL and pTau181, (Barro et al., 2020; Gaetani et al., 2019) and there is evidence to support male sex is associated with higher NfL levels (Mielke, 2020).

Dichotomized categories were established for the insomnia severity and cytoskeleton proteins. ISI categories were based on meeting the Minimal Clinical Important Difference (MCID) criteria, which is at least a 7-point difference (Morin et al., 2011). The categories were termed “met MCID” or “did not meet MCID”. The cytoskeleton protein categories were termed “responder” meaning that there was a decrease in the protein level for the absolute value change between blood draws. The second category termed “non responder” indicates that the protein level increased for the absolute value change between blood draws. A McNemar analysis was conducted using these categories to evaluate how homogenous the categories are.

## RESULTS

### Demographics

Forty individuals enrolled in the parent study. Eight individuals dropped out of the study due to conflict with the timing of the intervention or their sleep improved during the waiting period. Four participants opted out of the blood draw portion due to distance from the blood draw site. A total of 28 individuals were enrolled in this study between April 2021 and July 2022. The mean age for the sample is 41 years old (SD 13 years) with 22 women and six men (Table 1). Seventy-five percent of individuals in the sample experienced only one concussion, and 64% of the sample reported the cause of their concussion to be a motor vehicle accident. Seventy-eight percent of the sample reported they started experiencing difficulty with their sleep after their concussion. Demographic characteristics of the study participants are summarized in Table 1.

**Table 1:** Demographics

Mean Age (SD)	41(13)
Category	n
*Sex*	
Female	26
Male	6
*Education*	
Less than High School	1
GED	3
Associate degree	5
Some Collage	6
Bachelor	6
Masters	6
Doctorate	5
*Ethnicity*	
Non-Hispanic or Latino	28
Hispanic or Latino	3
Unknown	1
*Race*	
White	30
African American	2
*Marital Status*	
Single never married	6
Living with partner, not married	4
Married	14
Divorced	8
*Number of concussions*	
One concussion	22
Two concussions	2
Three concussions	4
Four concussions	4
*Source of concussion*	
Fall	8
Motor vehicle accident	20
Sport related	2
Work related	7
Other	4
*When did sleep issues start?*	
Before concussion	6
After concussion	26

### Neurofilament light

**Fig. 2:**
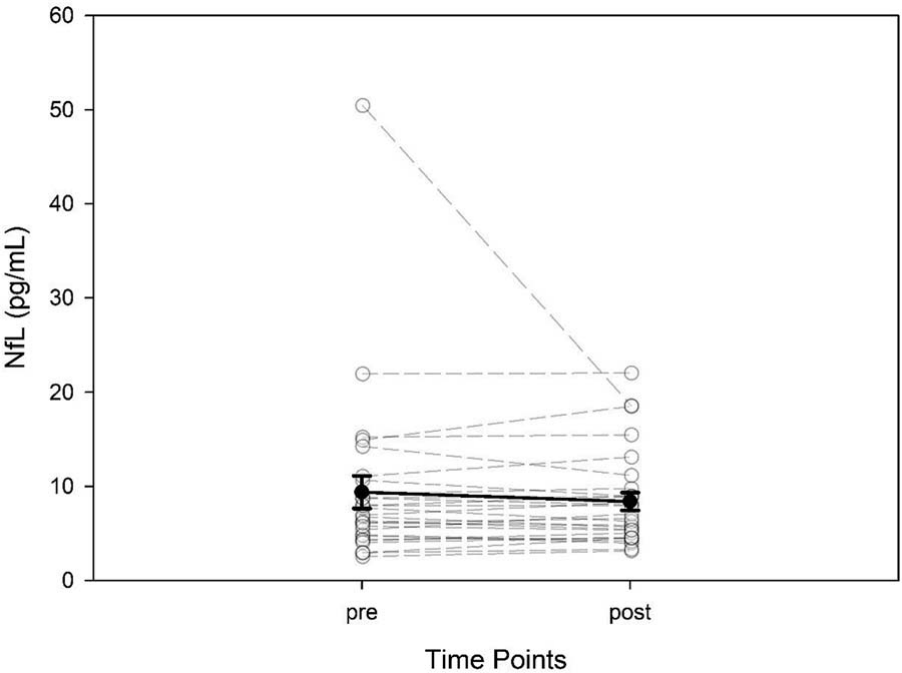
Change in NfL before and after CBT-I. Solid line is mean; dotted lines are individual values.

There was no significant change in NfL level from baseline to post intervention (*P* = 0.785; effect size 0.059). Post-intervention ISI score did not predict post-intervention NfL level (R^2^ 0.030; *P* = 0.376). When included in the model, higher baseline NfL value (*P* = 0.001) and older age (0.007) was predictive of a higher post-intervention NfL value, but post-intervention ISI (*P* = 0.709) and sex were not (*P* = 0.487). (Table 1; Fig. 2).

The results from McNemar analysis had a test statistic of 13 and a *P* value > 0.001. This means that more people met the MCID for insomnia without decreasing the NfL value compared to the number of people who had a decrease in NfL value and did not meet the MCID for insomnia. Twenty-five (89%) of participants met the MCID for insomnia, and 12 of out of 25 participants who met MCID for insomnia had a reduction of NfL (42%). There were three (10%) participants that did not meet MCID and increased their NfL values (Table 3).

One participant had a baseline NfL level of 50.44 pg/ml which is higher than the established cut off for the participant’s age (15 pg/ml) (Simrén et al., 2022). Following the intervention, this participant had a NfL value of 18.55 pg/ml which is a decrease of 63%. Demographically, this participant was a 59-year-old man with a history of three concussions. His baseline ISI value was 16 and following the CBT-I intervention the ISI score was 3.

### Phosphorylated tau

**Fig. 3:**
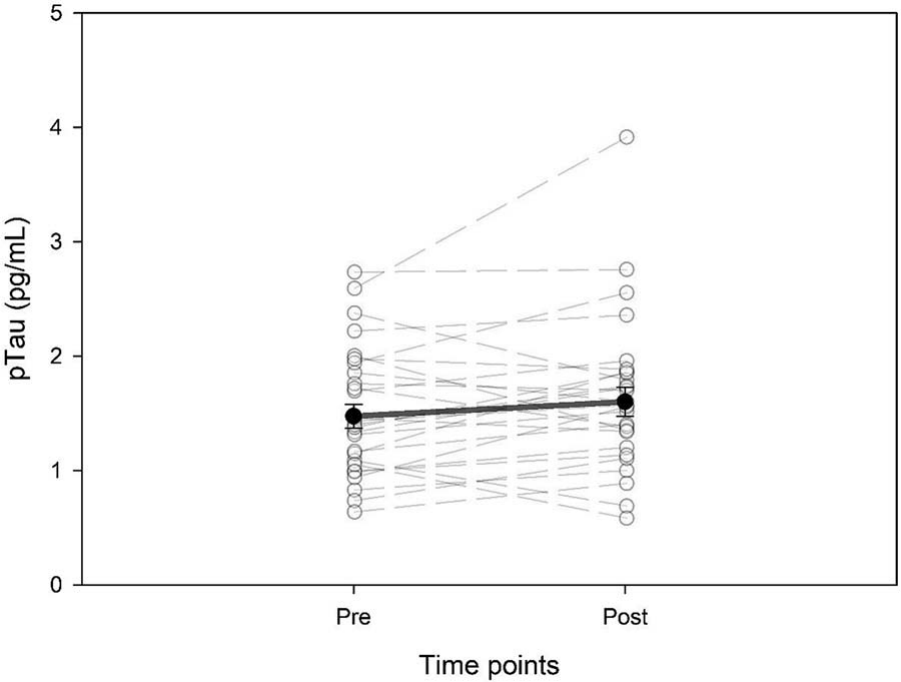
Change in pTau before and after CBT-I. Solid line is mean; dotted lines are individual values.

There was no significant change from baseline to post intervention (*P* = 0.133; effect size 0.28). Post-intervention ISI did not predict post-intervention pTau181 (R^2^ 0.023, *P* = 0.537). When included in the model, baseline pTau181 was a significant predictor of post-intervention pTau181 (R^2^ = 0.586, *P* < 0.001) but post-intervention ISI (*P* = 0.399) and sex were not (*P* = 0.457). (Table 2; Fig. 3).

**Table 2:** Linear Regressions for all study variable

	**Unstandardized Coefficients**		**Standardized Coefficients**			
	**B**	**SE**	**β**	**t**	**Sig. (p)**	**R** ^**2**^
**Model 1**						
Post-intervention ISI	0.220	0.245	0.174	0.901	0.376	−0.007
**Model 2**					<0.001	−0.717
Sex	−0.940	1.331	−0.079	−0.706	0.487	
NfL at baseline	1.004	0.413	0.284	2.432	0.001	
Age	0.469	0.519	0.133	0.904	0.007	
Post-intervention ISI	−1.260	0.551	−0.368	−2.285	0.709	
**Model 3**						
Post-intervention ISI	−0.021	0.034	−0.122	−0.626	0.537	−0.023
**Model 4**					<0.001	0.586
Sex	−0.178	0.236	−0.109	−0.756	0.457	
Ptau 181 at baseline	1.076	0.188	−0.879	5.732	<0.001	
Age	−0.007	0.008	−0.139	−0.951	0.352	
Post-intervention ISI	−0.020	0.023	−0.114	−0.860	0.399	

A Linear Regression Models (Models 1 and 2), Dependent variable NfL post CBT-I intervention. (Models 3 and 4) Dependent variable pTau181 post CBT-I intervention.

**Table 3:** McNemar analysis for MCID on ISI and values of NfL. MCID on the ISI is a decrease of 7 on the ISI. Responder is denoted as a decrease of NfL levels following the CBT-I intervention

	NfL Responder	NfL non-responder	McNemar Test statistic χ^2^ (p value)
Met MCID on ISI	12	13	
Did not meet MCID on ISI	0	3	
χ^2^			0.000311491 (>0.001)

ISI: Insomnia Severity Index; MCID: Meaningful Clinical Important difference; NfL: Neurofilament Light

The McNemar results for pTau181 had a test statistic of 13.235 and *P* value > 0.001. More participants met the MCID on insomnia without decreasing the pTau181 values compared to the number of people who had a decreased the pTau181 values and not having meeting the MCID criteria for insomnia. Twenty-five (89%) of participants had met the MCID criteria on the ISI and nine of participants met the MCID criteria and had a reduction in the pTau181 level (32%). Two (7%) of participants did not meet the MCID criteria and did not reduce their pTau181 level. (Table 4).

One individual had a 44% decrease in pTau181. This individual was a 50-year-old woman with a history of three concussions. Her starting pTau was 1.05 pg/ml and following the CBT-I intervention her pTau181 decreased to 0.58 pg/ml. Her insomnia baseline value was 18 and decreased to 9 following the CBT-I intervention.

## DISCUSSION

This is the first study to examine if a behavioral intervention to enhance sleep will improve NfL and pTau181 in individuals with a concussion. Counter to our hypothesis, there was no significant within group change for improvement in protein levels. Furthermore, post-intervention insomnia severity was not predictive of post intervention NfL or pTau181. However, approximately half of the participants demonstrated a reduction in NfL and pTau181 levels following CBT-I as well as clinically meaningful improvement in insomnia severity. Future studies are needed to examine factors or combination of factors that contribute to improvement or lack thereof in biomarkers indicating neuronal damage. Based on responders demonstrating a meaningful improvement in insomnia severity, sleep enhancement remains a worthwhile factor to continue to explore.

Counter to our hypothesis, there was no significant within group change in the cytoskeleton proteins. This may be largely due to many participants starting the study with protein levels within a normal range, thus limiting the opportunity for change. The reason many participants’ protein levels were within normal range despite having a concussion may be due to the fact that most of the participants were younger individuals; young adults typically have lower amounts of NfL and pTau181 in the blood stream (Gaetani et al., 2019; Thebault et al., 2020). The lower protein levels in younger brains may be because of the resiliency and efficiency in healing after the concussion injury. Another explanation is that this study is exploratory and could be underpowered limiting the ability to detect change (Kinney et al., 2020). Lastly, all participants were at least 4 weeks post their concussion injury, but we did not assess the exact time since concussion which may impact interpretation due to brain healing that has already occurred. Anecdotally, many participants reported being at least 3 months to 1 year post their injury indicating the sample were likely in the chronic stage of concussion rather than subacute. It has been established that over time NfL values are higher closer to the injury and then stabilize or decrease in a linear fashion years after the concussion injury although Tau levels were more variable across time in the 5 years post-concussion (Shahim et al., 2020b). It may be that intervening to address sleep disturbances in the acute state of concussion may have more impact on influencing NfL and pTau181 or it may be that people with higher protein levels will be more likely to benefit from sleep enhancement to mitigate protein levels.

**Table 4:** McNemar analysis for MCID on ISI and values of pTau181

	pTau181 Responder	pTau181 non-responder	McNemar Test statistic χ^2^ (p value)
Met MCID on ISI	9	16	
Did not meet MCID on ISI	1	2	
χ^2^			13.035 (>0.001)

MCID on the ISI is a decrease of 7 on the ISI. Responder is denoted as a decrease of pTau181 levels following the CBT-I intervention. ISI: Insomnia Severity Index; MCID: Meaningful Clinical Important difference; pTau181: phosphorylated Tau 181

Interestingly, there were individuals who had an MCID on the ISI and a reduction in both their cytoskeleton protein levels. It is unknown why some people met the MCID and lowered their protein level and why others did not. It is possible that there could be a certain criterion of participant to elicit the desired effect of both reducing insomnia symptoms and cytoskeleton protein levels. This criterion may be related to the number of concussions, time since concussion, age, and history of insomnia. It has been established that age is associated with higher levels of both NfL and pTau181 (Barro et al., 2020; Gaetani et al., 2019; Thebault et al., 2020). Additionally, there is growing evidence that time since injury and history of concussion factor into the levels of NfL and Total Tau (McDonald et al., 2021; Shahim et al., 2014; Shahim et al., 2020a; Shahim et al., 2020b; Zetterberg et al., 2013). Therefore, there is an opportunity to further characterize this relationship between insomnia and cytoskeleton proteins in individuals with a concussion.

### Limitations and future directions

This is an exploratory study with limitations to acknowledge. One limitation is that the current study had a small sample size which limits the interpretation of the results and may lead to type II error. Another limitation is this study included more women than men that could limit the generalizability of the results as it is still debated how sex effects levels of NfL and pTau181. The age range of the sample ranged from 18–64 with the majority of the sample falling into the ages of 20–35, limiting the understanding of how older age and a concussion impacts NfL and pTau181 values. Also, it is unknown the time since injury limiting the ability to capture NfL and pTau181 at their peak release.

Future studies should continue to explore if sleep enhancement contributes to reduction of neuronal injury, specifically what factor(s) influence who is a “responder”. Future research should consider how time since injury may influence sleep’s impact on protein levels. In addition, future research may consider examining sleep enhancement intervention in older adults as they may start at elevated protein levels and thus be more amenable to reduction of protein levels (Brickman et al., 2022; Scherling et al., 2014; Teunissen et al., 2022; Zhou et al., 2022). Future studies should also consider collecting NfL and pTau181 samples immediately after concussion to a longer-term follow-up and pairing the sampling with assessments for insomnia for better understanding how levels change over time and the relationship between sleep and cytoskeleton proteins.

## CONCLUSIONS

This is the first paper that has examined the impact of a sleep intervention on neuronal injury biomarkers in individuals following a concussion. While there were no group level improvements in NfL or pTau181, about half the participants demonstrated improvements in protein levels and insomnia severity. This lays groundwork for continuing to explore the relationship between sleep and NfL and pTau181.

## Data Availability

Data will be shared upon request to the corresponding author.
